# Assessing the Impact of Persistent HIV Infection on Innate Lymphoid Cells Using In Vitro Models

**DOI:** 10.4049/immunohorizons.2300007

**Published:** 2023-03-31

**Authors:** Aude Boulay, Sara Trabanelli, Stéphanie Boireau, Myriam Boyer-Clavel, Sébastien Nisole, Pedro Romero, Camilla Jandus, Anne-Sophie Beignon, Nathalie J. Arhel

**Affiliations:** * Viral Trafficking, Restriction and Innate Signaling, Institut de Recherche en Infectiologie de Montpellier (IRIM), Université de Montpellier, CNRS, Montpellier, France; † Department of Pathology and Immunology, Faculty of Medicine, University of Geneva, Geneva, Switzerland; ‡ Ludwig Institute for Cancer Research, Lausanne Branch, Lausanne, Switzerland; § Montpellier Ressources Imagerie, Biocampus, Université de Montpellier, CNRS, Montpellier, France; ¶ Department of Oncology, University of Lausanne, Épalinges, Switzerland; ‖ Université Paris-Saclay, Inserm, CEA, Center for Immunology of Viral, Auto-Immune, Hematological and Bacterial Diseases (IMVA-HB/IDMIT), Fontenay-aux-Roses, France

## Abstract

Pathogens that persist in their host induce immune dysfunctions even in the absence of detectable replication. To better understand the phenotypic and functional changes that persistent infections induce in sentinel innate immune cells, we developed human PBMC-based HIV models of persistent infection. Autologous nonactivated PBMCs were cocultured with chronically infected, acutely infected, or uninfected cells and were then analyzed by unsupervised high-dimensional flow cytometry. Using this approach, we identified prevalent patterns of innate immune dysfunctions associated with persistent HIV infections that at least in part mirror immune dysfunctions observed in patients. In one or more models of chronic infection, bystander CD16^+^ NK cells expressing markers of activation, such as CD94, CD45RO, CD62L, CD69, CD25, and immune checkpoints PD1, Tim3, TIGIT, NKG2A and Lag3, were significantly reduced. Conversely, helper ILC subsets expressing PDL1/PDL2 were significantly enriched in chronic infection compared with either uninfected or acute infection, suggesting that chronic HIV-1 infection was associated with an inhibitory environment for bystander ILC and NK subsets. The cell-based models of persistent infection that we describe here provide versatile tools to explore the molecular mechanisms of these immune dysfunctions and unveil the contribution of innate immunity in sustaining pathogen persistence.

## Introduction

Persistent viral infections, which arise when the host immunity fails to clear the invading pathogen, are chronic diseases that occasionally require lifelong treatment (1). Despite the lack of any clinical symptoms and the absence of detectable viral replication, evidence suggests that latent infection drives immune dysfunctions and overall activation, which can lead to increased comorbidity and a risk of cancer several decades after initial infection (2–4). Latent herpesvirus infection, for instance, has been reported to alter the host immune response to other Ags and to exacerbate seemingly unrelated diseases (5). Recently, persistent infection with SARS-CoV-2 was also described and linked to long COVID-19 (6).

Patients infected with HIV and receiving effective antiretroviral therapy (ART) also have the characteristics of a persistent infection. Although ART efficiently suppresses viral replication, HIV persists as a latent integrated provirus in target cells. Patients have an increased risk for comorbidities, immune dysfunction, and inflammation, as evidenced by high levels of serum immune activation markers, inflammatory cytokines, and activated immune cells (7).

HIV-1 is a positive-strand RNA virus, belonging to the family of *Retroviridae*, that targets CD4^+^ T cells and other CD4-expressing cells, such as macrophages, dendritic cells, and monocytes. HIV-1 requires reverse transcription of its RNA genome into dsDNA and integration in the host chromatin to replicate. Latency occurs when a productively infected cell reverts to a resting state that is nonpermissive for viral gene expression (8). In this latent state, the virus persists essentially as genetic information, and infected cells are not efficiently targeted by the immune system or ART.

HIV infection triggers the infected cell’s intrinsic immunity in multiple ways that involve the detection of pathogen-associated molecular patterns by pattern recognition receptors (9). During the early phase of infection, incoming viral RNA can trigger TLR7 (10), whereas reverse-transcribed intermediates and incoming viral capsid can activate cyclic GMP–AMP synthase (11–15). In the late phases of the replication cycle, HIV-1 genomic RNA can activate RIG-I signaling (16–18). The recognition of viral components triggers the release of IFN, damage-associated molecular pattern (DAMP) molecules, and other proinflammatory mediators that activate innate immune cells. In the absence of viral replication, one would expect that the innate immune system is no longer triggered. However, even during successful ART, latent HIV continues to fuel chronic immune activation and inflammation (19, 20). Latently infected cells still contain the integrated provirus and express viral transcripts and proteins at low levels (21) that can continue to spread danger signals and proinflammatory mediators to bystander innate immune cells (22–24).

The Innate immune system is composed of a complex network of cells that also sense pathogens and release soluble mediators that exert potent antiviral activity and contribute to activating and recruiting other cells. They also mediate other innate functions concurring to pathogen clearance such as pathogen phagocytosis, killing and cytotoxicity (25). Among these, innate lymphoid cells (ILCs) are non-T, non-B lymphocytes comprising cytotoxic NK cells and helper ILCs, a recently identified type of helper effector innate immune cells (26–28). Although ILCs lack Ag-specific receptors, they express a broad array of cell surface and intracellular receptors that allow them to sense changes in their microenvironment and calibrate their cytokine secretion in response to pathogenic infections and tissue damage. Helper ILCs are divided into three distinct subsets, ILC1, ILC2, and ILC3, that mirror the functional specialization of adaptive helper CD4 T cells.

Several studies have reported perturbations in the innate lymphoid compartment during progressive HIV/SIV infection, including a drastic and irreversible depletion of ILCs from mucosae, blood, and lymph nodes during acute infection (29–33). Similar depletions in ILCs have also been reported in patients receiving successful ART (29). Notwithstanding the fact that, unlike CD4^+^ T cells, ILCs are not recovered by ART unless treatment is initiated during the acute early phase (29, 30, 34), the alteration of ILCs even after at least 12 y of suppressive ART (35) is concordant with an underlying defect, such as HIV-associated chronic immune activation (36), which may prevent the optimal recovery of ILC functions. Therefore, one hypothesis is that during persistent infections, ILC functions might be compromised by low-level DAMPs or activatory signals. Testing this in human cohorts is challenging because it is not possible to conclude whether ILC functions are compromised by the persistent integrated provirus per se or by unrelated factors such as antiretroviral drug toxicity or traditional risk factors that are prevalent in patients with HIV, such as substance abuse, obesity, and hypertension.

To address how persistent infection alters ILCs, we devised a human PBMC-based coculture model allowing us to examine functional and phenotypic changes in ILCs upon coculture with autologous cells harboring integrated nonreplicating virus or acutely infected with HIV. Using multiparametric flow cytometry and gating exclusively on bystander uninfected cells, we performed comparative in-depth profiling using 16-color flow cytometry of blood ILCs.

## Materials and Methods

### PBMC isolation and coculture conditions

Buffy coats from healthy donors were obtained from the Etablissement Français du Sang (Toulouse, France). PBMCs were isolated by centrifugation on Ficoll gradient, counted, and batch frozen at 50 × 10^6^ cells/vial.

On day 0 (d0), PBMCs were thawed and immediately activated with PHA (1 µg/ml) for 24 h and stimulated with IL-2 (10 ng/ml) for a further 3 d. On d3, stimulated PBMCs (5 × 10^6^ for each condition) were confirmed to be >90% viable. Cells were then infected with wild-type or env^−^ HIV-1 or transduced with lentiviral vector (LV) at 0.5–1 ng p24 per 1000 cells with 10 µg/ml DEAE dextran. After 2 h, RPMI and IL-2 (10 ng/ml) were added. Wild-type virus (acute and ART conditions) were NL4-3-based molecular clones encoding internal ribosome entry site (IRES)–enhanced GFP (eGFP) in the *Nef* open reading frame. The env^−^ virus contained a frameshift in the *env* coding region. The HIV-1-derived vector (LV condition) contained an internal CMV promoter to drive eGFP expression. Both the env^−^ virus and HIV vector were pseudotyped with a vesicular stomatitis virus G envelope.

On d6, cells were washed twice to eliminate residual vector/virus particles, and the percentage of eGFP-positive cells was assessed by flow cytometry. Autologous unstimulated PBMCs were then thawed, counted, and cocultured with transduced/infected or uninfected PBMCs at a 1:1 ratio. In the ART model, antiviral compounds were added at this time point. On d9, cocultures were washed and split into six labeling panel conditions with 1.5–2 × 10^6^ cells per panel.

Experiments were performed independently for each chronic infection model (LV, ART, env^−^), each with the corresponding uninfected and replicative HIV control conditions. Each experiment was performed at least twice on different donors.

### Vectors and viruses

The HIV-1-derived vector LV-eGFP was produced by cotransfection of the pTRIP-CMV-eGFP (37), 8.74 encapsidation, and vesicular stomatitis virus G envelope expression plasmid pHCMV-G plasmids in HEK-293T cells. The replicative virus HIV1-eGFP was produced by transient transfection of HEK-293T cells using calcium phosphate precipitation with a pBR-NL43-IRES-eGFP-env^+^nef^+^ construct (38). Vector or virus particles were harvested at 48 h after transfection, filtered on a 0.45-µm filter, and titered by a p24 (CA) ELISA according to the manufacturer’s instructions (Clontech). All viral molecular clones encoded IRES-eGFP in the *Nef* open reading frame, thus allowing us to monitor productively infected cells. Viruses were titered by ELISA against the p24 capsid Ag (Takara).

### Abs and flow cytometry acquisition

All experiments were compensated using autologous PBMCs labeled with a single fluorophore Ab. Moreover, Abs (from BioLegend, Miltenyi, BD Biosciences, and eBioscience; Supplemental Table I) were titrated on activated PBMCs prior to the experiments to determine the optimal concentration yielding the greatest signal for the positive population and the lowest background signal for the negative population. Some Abs were not possible to titer on PBMCs because of the low frequency of their Ags and were used at the dose of 2 µl. The stain index was obtained as follows: 50 µl PBMCs containing 1 × 10^6^ cells were labeled with 50 µl diluted Ab for 20 min at room temperature. Cells were then washed and fixed in 1% paraformaldehyde for 30 min before washing again. Cells were acquired on a BD LSRFortessa equipped with four excitation lasers (405, 488, 561, and 633 nm) using the BD FACSDiva software program. The stain index Δ was obtained using the equation Δ = (MFI pos – MFI neg)/2 × SD, where MFI is the mean fluorescence intensity and SD is the standard deviation.

### Panels and labeling conditions

All samples were stained and acquired immediately on d9. All labeling panels contained lineage markers and viability dye in the 488/530 channel. As such, all FITC/eGFP^+^ events were Lin^+^, nonviable, HIV-1 infected, or a combination of all and were excluded from the analysis. Flow cytometry using 16-parameter acquisition was performed on a BD LSRFortessa flow cytometer, and analyses were performed using FlowJo.

### Gating strategy

The frequency of live cells was assessed using 488/520 Viability Fixable Dye and was similar between the different coculture models (above 85%). Total ILCs, defined as lineage negative (Lin^−^) (CD3^−^, CD4^−^, CD14^−^, CD15^−^, CD19^−^, CD20^−^, CD33^−^, CD34^−^, CD203c^−^, FcεRI^−^) CD16^−^ CD56^−^ CD8^−^ CD127^+^ cells, were, on average, 0.5% of the total live PBMCs. ILC precursors (ILC3/ILCP) were further defined as cKit^+^ (CD117^+^) CRTH2^−^ cells, whereas cKit^−^ CRTH2^−^ cells were gated as ILC1, and CRTH2^+^ cells were gated as ILC2.

### Flow cytometry pipeline and analyses

Samples were acquired at continuous medium flow on a BD LSRFortessa flow cytometer. FlowJo version 10 and the plugin FlowSOM (Self-Organizing Map) installed in R were used for data downscaling, clustering, analyses, and visualization. The compensation matrix was generated on the basis of single-marker samples that were acquired for each experiment (see Abs used for compensation in column J of Supplemental Table I). The same operating procedure was applied for sample collection and processing across samples. Unsupervised analysis combining dimension reduction (*t*-distributed stochastic neighborhood embedding [tSNE]) followed by clustering (FlowSOM) was performed on Lin^−^ cells using all markers except lineage as follows: After data compensation, the expression values were transformed using an inverse hyperbolic sine (arcsinh) transformation and data were cleaned for signal quality using FlowClean and downsampled using the DownSample plugin so that concatenated samples had the same absolute number of cells.

## Results

### Cell-based models of persistent HIV infection

**FIGURE 1.**
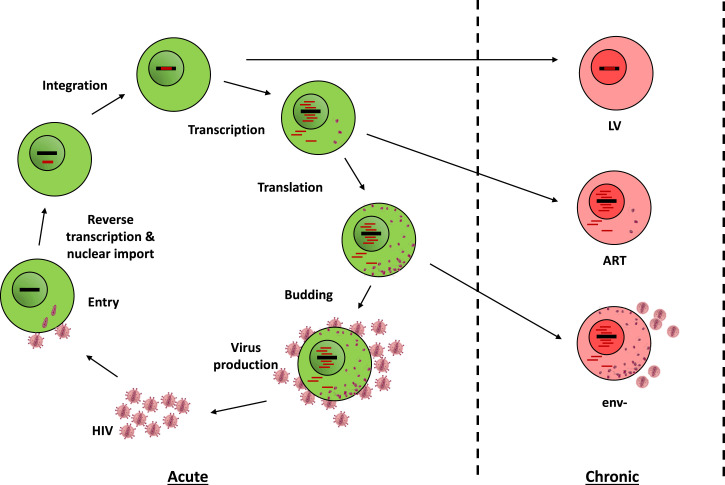
Schematic representation of the HIV-1 replication cycle in CD4^+^ T cells (acute model) and of the three cell-based models of persistence (chronic models), which were compared with uninfected cocultures.

To understand how chronic HIV infection modulates innate lymphocytes, we devised primary PBMC-based models of persistent HIV infection. In these models, uninfected or acutely or chronically infected PBMCs are cocultured with autologous unstimulated PBMCs from healthy donors for 3 d, after which bystander ILCs are analyzed for functional and phenotypic markers. We generated three different models of HIV-1 persistent infection based on primary human PBMCs compared with acute infection and no infection (Fig. 1). The three models were designed to best reproduce the different viral pathogen-associated molecular patterns and cellular disruptions that persist in HIV-infected cells after acute infection, namely genomic integration and the presence of viral transcripts and proteins in the cytoplasm.

In the first model, LV, PBMC infection with replicative HIV-1 (a model of acute infection) was compared with single-cycle infection with a self-inactivating HIV-1 vector. This vector lacked all HIV genes but contained the *cis*-acting regulatory elements and long terminal repeat regions necessary for integration. All the required viral proteins were provided in *trans* on a packaging plasmid during vector particle production. In this model, persistent infection is mimicked by allowing viral genome integration in the host chromatin while preventing subsequent expression of viral transcripts, which essentially mimics cells harboring integrated proviruses that are defective or blocked at the transcriptional level (39, 40) (Fig. 1).

In the second model, ART, acute infection is allowed to proceed for 3 d to establish a viral reservoir and is then subjected to treatment with a reverse transcriptase inhibitor (nevirapine 5 µM) and an integration inhibitor (dolutegravir 0.5 µM) to mimic therapeutic viral suppression in patients. In this model, the viral genome that integrated prior to ART can be transcribed and generates de novo particles; however, these will fail to productively infect new target cells (Fig. 1).

In the third model, env^−^, chronic infection is mimicked by infecting cells with envelope-defective HIV-1, which can also be transcribed but does not yield infectious particles. The difference between the ART and env^−^ models is that ART is administered after initial viral spread and will halt HIV replication cycles at multiple steps, hence creating a heterogeneous pattern as observed in patients, whereas infection with env^−^ virus will not spread and will halt infection in a synchronized manner (Fig. 1).

### Phenotypic characterization of sentinel ILCs

**FIGURE 2.**
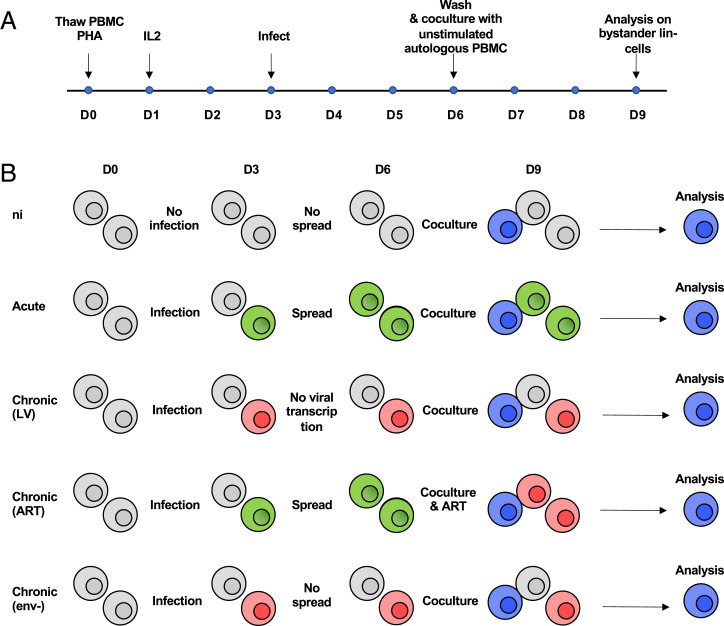
(**A**) Timeline of the experimental protocol. (**B**) Schematic representation of the different PBMC coculture conditions involving uninfected cells (gray), cells infected with replicating HIV-1 (green), and HIV-1-infected cells without ongoing replication (red) cells. Bystander Lin^−^ cells (blue) were analyzed by 16-color flow cytometry after 3 d of coculture.

To determine whether persistently infected cells can alter bystander ILCs, unstimulated PBMCs were cocultured with chronically infected autologous PBMCs for 3 d, then labeled with phenotypic and activation markers (Fig. 2A, 2B). A total of 53 markers, 17 to identify NK and ILC subtypes and 36 to phenotypically characterize them, were subdivided into 6 different panels (Supplemental Fig. 1A) for analysis by 16-parameter flow cytometry. Lineage markers and a stain for dead cells were all FITC labeled, as a dump channel, together with productively infected cells (which are eGFP^+^). This gating step selected ∼10% uninfected lineage-negative (Lin^−^) cells, which is concordant with their reported frequency in peripheral blood (41).

ILCs (including NK cells) are Lin^−^ and therefore were not labeled by any of the FITC-conjugated Abs. NK cells were defined on the basis of their expression levels of CD16 and CD56, followed by CD8 and CD94 (Supplemental Fig. 1B). Helper ILCs express the IL-7 receptor (CD127) and are further subdivided into ILC1, ILC2, and ILC3 on the basis of their surface expression of CRTH2 (chemoattractant receptor homologous molecule expressed on Th2) and cKit (stem cell growth factor receptor, tyrosine-protein kinase Kit, also called CD117). Because experiments were performed on thawed PBMC samples, we assessed that ILC analyses on frozen material provided consistent and reproducible results across donors. To validate our strategy, manual gating was performed to identify the different ILC subsets (Supplemental Fig. 1C). In three different donors, ILCs represented ∼0.1% of live PBMCs. Furthermore, ILC1 represented ∼40% of all ILCs, then 15–20% for ILC2 and ILC3. This is consistent with the reported frequency of helper ILCs in circulating PBMCs (41).

To achieve high-dimensional analysis and bypass manual gating, which is error-prone and time-consuming, we opted for an unsupervised analysis of ILC and NK cell subsets from the three different models of chronic infection and compared these with the uninfected condition and acute infection. Samples from each chronic experimental condition (LV, ART, or env^−^) were concatenated with their uninfected and acutely infected controls, and Lin^−^ cells were analyzed by tSNE dimensionality reduction.

Using this approach, we excluded Lin^+^ cells and used ILC- and NK-specific markers to simultaneously identify three different ILCs (ILC1, ILC2, ILC3/ILCP) (42) and two different NK cell populations (Supplemental Fig. 1B), CD56^dim^CD16^+^ and CD56^bright^CD16^−^ cells. ILCs that were CD127^dim/−^ were referred to as “unconventional ILCs” (43).

**FIGURE 3.**
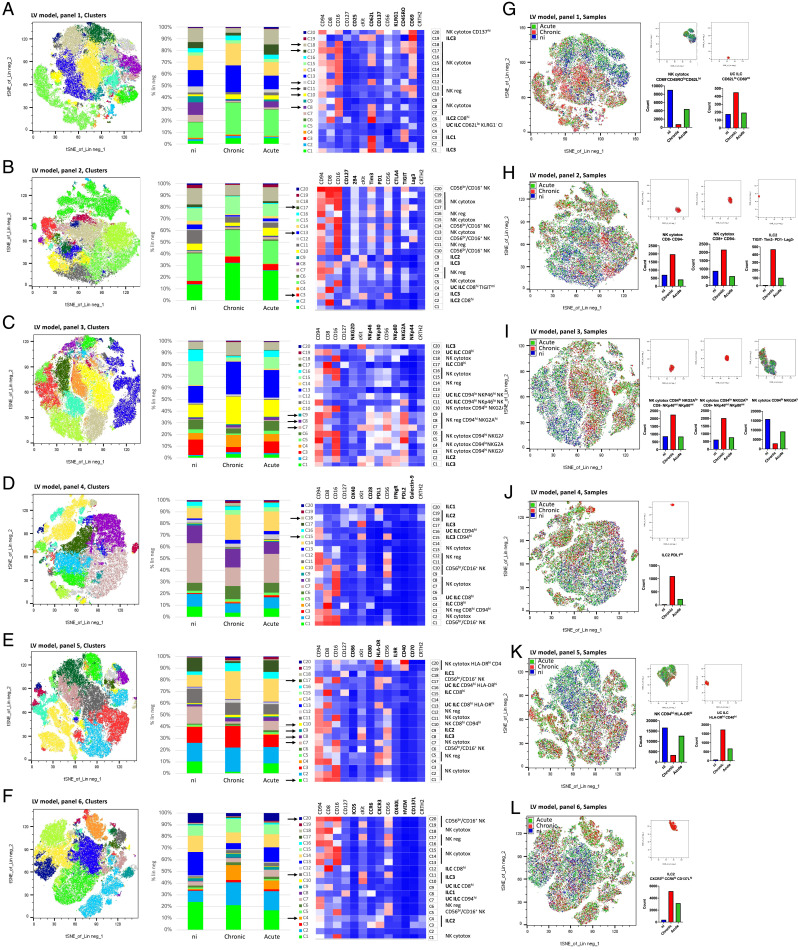
tSNE dimensionality reduction analysis of PBMCs from the LV model coculture. tSNE plots from a representative experiment are colored to indicate the 20 clusters (**A**–**F**) or the three samples (ni, acute and chronic; **G**–**L**) for an individual donor. tSNE plots correspond to Ab panels (Supplemental Fig. 1A) 1 (A and G), 2 (B and H), 3 (C and I), 4 (D and J), 5 (E and K), and 6 (F and L). Clusters are quantified as the percentage of Lin^−^ and labeled by manual cell type annotation based on the seven markers common to all panels. UC, unconventional ILC (CD56^−^CD16^−^CD127^−^); Unchar., uncharacterized, negative for all tested markers. Clusters that are discussed in the *Results* section are pinpointed by a black arrow.

The FlowSOM analysis was set to identify 20 clusters (C1–C20), which allowed all major ILC subtypes to be identified without redundancy or unnecessary complexity. The relative expression of all 13 surface markers within each cluster was represented as heat maps, and major ILC subsets were annotated manually on the basis of expression of the seven markers shared between Ab panels (Fig. 3, Supplemental Figs. 2 and 3). The unsupervised analysis identified CD56^dim^CD16^+^ (NK cytotox) and immunoregulatory CD56^bright^CD16^−^ (NK reg) cells, as well as ILC1, ILC2, and ILC3/ILCP. Additionally, the unsupervised analysis identified clusters that were CD56^−^CD16^−^CD127^−^, referred to as unconventional ILCs and an unconventional CD56^bright^/CD16^+^ NK subset (CD56^br^/CD16^+^ NK).

### Prevalent changes in the expression profiles of sentinel ILCs upon coculture with HIV-infected cells

In the LV model, coculture with HIV-infected cells (chronic or acute) led to an overall increase of relative abundance in CD94^hi^ regulatory NK cells and ILCs (two- to threefold), which were also CD69^hi^ NKG2A^hi^ NKp46^hi^ CXCR3^hi^, but CD45RO^lo^ CD62L^lo^, and negative for immune checkpoint (ICP) molecules Tim3, T cell immunoreceptor with Ig and ITIM domains (TIGIT), Lag3, and PDL1 (C10, Fig. 3A; C3, Fig. 3B; C7–C9, Fig. 3C; C15, Fig. 3D; C8–C10, Fig. 3E; C4, C11, Fig. 3F).

In the ART model, we also observed a weak overall increase (two- to threefold) in CD94^hi^ CD69^hi^ NKp80^hi^ or CD8^hi^ regulatory NK cells and ILCs in both HIV-infected cocultures (C12, Supplemental Fig. 2A; C6, Supplemental Fig. 2C; C10, Supplemental Fig. 2E). Conversely, there was an overall decrease in cytotoxic NK cells and ILCs expressing ICP molecules Tim3, TIGIT, PDL1 and PDL2, and NKp80 (C8 and C19, Supplemental Fig. 2B; C18, Supplemental Fig. 2C; C4 and C8, Supplemental Fig. 2D; C17, Supplemental Fig. 2E; C2 and C11, Supplemental Fig. 2F) in both chronic and acute conditions.

In the env^−^ model, coculture with HIV-1-infected cells, either wild type or env^−^, led to an increase in the proportion of cytotoxic NK cells expressing CD69 compared with coculture with uninfected cells (4–6-fold increase in CD69^hi^ C13, C15, and C16 and 8–18-fold decrease in CD69^lo^ C5 and C12; Supplemental Fig. 3A). Both wild-type and env^−^ infections also led to an increase in CD94-expressing cells, which also expressed TIGIT (four- to fivefold increase in C9, Supplemental Fig. 3B), NKp46, NKp80, and NKG2A (threefold increase in C11, Supplemental Fig. 3C). On the whole, few phenotypic differences were uncovered between cells cocultured with either wild-type and env^−^ HIV-1-infected cells, which differ only in the presence of infectious particles in the supernatant. This suggests that the phenotypic changes that were detected in bystander ILC and NK subsets do not require extracellular infectious virions.

In conclusion, the unsupervised analysis uncovered substantial differences in the cellular phenotypes of NK cells and helper ILCs, highlighting the strong capacity of ILCs to sense their environment (Fig. 3, Supplemental Figs. 2 and 3). Coculture with HIV-1-infected cells led to an overall increase in bystander NK/ILC populations that expressed markers of maturation and activation, such as CD94, CD69, NKG2A, and CD8, and an apparent decrease in NK/ILC clusters that expressed ICP molecules, regardless of whether cells were chronically or acutely infected. This confirmed that the 3-d coculture protocol is sufficient to induce functional changes in bystander innate immune cells.

### Changes in the expression profiles specifically associated with the acute coculture condition

One feature that was unique to acute infection cocultures was a strong increase in the relative abundance of CCR6-positive ILC subsets (e.g., 125-fold increase in C4; Supplemental Fig. 3F, Table I). This increase was not observed in env^−^ virus samples and was reduced in ART-treated samples (C5, Supplemental Fig. 2F, 47-fold for the acute infection model compared with 31-fold for the ART coculture), suggesting that this signature is associated with the presence of de novo produced virus particles in the coculture supernatant. CCR6 is a chemokine receptor that mediates homing to tissues. An upregulation of CCR6 on NK subsets has been reported during acute SIV infection, which is then lost upon chronic infection (44).

**Table I.**
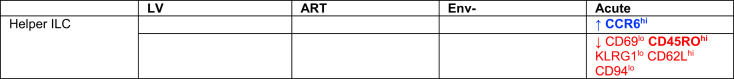
Changes in ILC subsets unique to the acute condition

Only changes that were above threefold relative to the uninfected condition (ni) are shown. An increase in the relative abundance of the cell subset is represented with an arrow pointing up, whereas a decrease is indicated by an arrow pointing down. Very brightly expressed Ags in a given cell subset are highlighted in bold. Helper ILCs include ILC1, ILC2, ILC3/ILCP, and unconventional ILCs.

### Changes in the expression profiles specifically associated with one or more chronic coculture condition(s)

One aim was to identify signatures that might be common to all three chronic infection models. The LV model highlighted several differences between the chronic and acute cocultures (Table II). Specifically, coculture with LV-infected cells was associated with a depletion of CD94^hi^ CD62L^hi^ cytotoxic NK cells (18- and 30-fold decrease in C8 and C12, respectively; Fig. 3A) as well as CD45RO^+^ CD69^hi^ cytotoxic NK cells (4-fold decrease in C18; Fig. 3A), CD8^hi^ CD94^hi^ cytotoxic NK cells (decrease in CD8^hi^ C12 and C17 relative to uninfected and acute conditions; Fig. 3A), and CD8^−^ CD94^hi^ cytotoxic NK cells (C13 and C17; Fig. 3B). These CD94^hi^ cells also expressed Tim3, TIGIT, and Lag3 (C17; Fig. 3B). There was a 27-fold increase in OX40^hi^ PDL1^hi^ ILC2 (C18; Fig. 3D). Together, these results suggest that coculture with LV-transduced cells causes reductions in the frequency of activated ILC and NK subsets.

**Table II.**
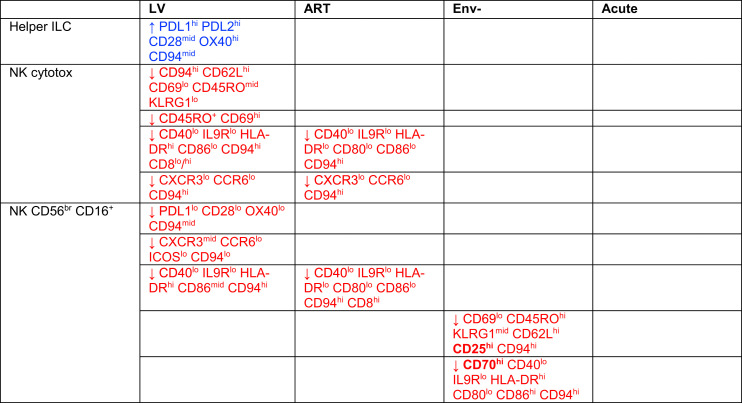
Changes to ILC subsets unique to one chronic condition or common to several chronic conditions

“Only changes that were above threefold relative to the uninfected condition (ni) are shown. An increase in the relative abundance of the cell subset is represented with an arrow pointing up, whereas a decrease is indicated by an arrow pointing down. Very brightly expressed antigens in a given cell subset are highlighted in bold. Helper ILCs include ILC1, ILC2, ILC3/ILCP, and unconventional ILCs.”

To confirm these changes, we colored the tSNE plots to specifically highlight differences between the chronic coculture model on the one hand and both uninfected and acute infection on the other (Fig. 3G–3L). These revealed a relative decrease in cytotoxic NK cells that were CD69^lo^ CD45RO^lo^ CD62L^hi^ CD94^hi^ NKG2A^hi^ HLA-DR^hi^ (Fig. 3G, 3I, and 3K) and an increase in unconventional ILCs that were CD62L^lo^ CD69^mi^ HLA-DR^hi^ CD40^hi^ (Fig. 3G and 3K), as well as CD94^lo^ NKG2A^lo^ cytotoxic NK cells and PDL1^hi^ ILC2 (Fig. 3H–3J, Table II).

In contrast, fewer changes were observed between replicative HIV-1 and either treatment with ART or infection with single-cycle env^−^ virus (Supplemental Figs. 2 and 5, Table II). These two chronic conditions also showed the most overlap with the acute condition in terms of changes in NK ILC subsets (Table III).

**Table III.**
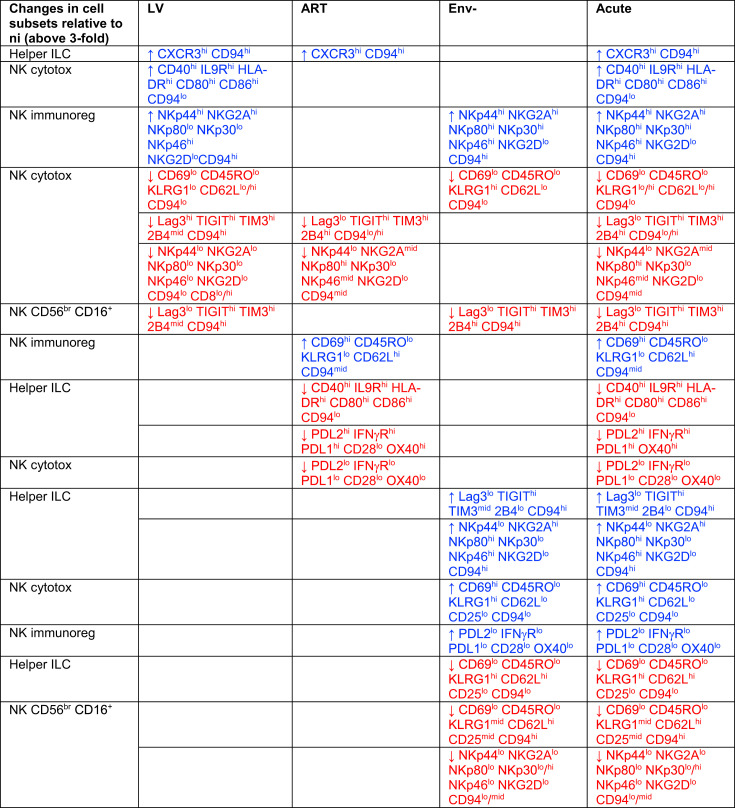
Changes in ILC subsets common to both chronic and acute conditions

“Only changes that were above threefold relative to the uninfected condition (ni) are shown. An increase in the relative abundance of the cell subset is represented with an arrow pointing up, whereas a decrease is indicated by an arrow pointing down. Helper ILCs include ILC1, ILC2, ILC3/ILCP, and unconventional ILCs.”

After unsupervised analysis of the data across three different models of chronicity, only a few markers showed consistent change across chronic conditions only. One feature was a relative decrease in CD56^br^/CD16^+^ NK cells, which was occasionally observed in both chronic and acute infection models (two- to threefold decrease in C1, Supplemental Fig. 2E; threefold decrease in C17, Supplemental Fig. 3B), but which was generally specific to the chronic condition (threefold decrease in C20, Fig. 3F; sixfold decrease in C15, Supplemental Fig. 3B), and associated with CD94^hi^ HLA-DR^hi^ expression (sevenfold decrease in C1, C7, and C17, Fig. 3E).

**FIGURE 4.**
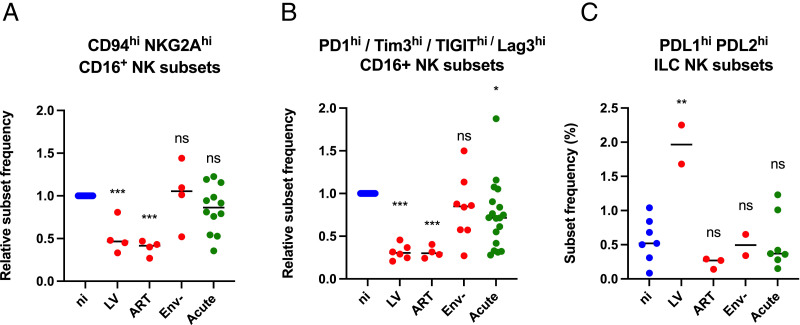
Summary of changes in the relative abundance of different ILC and NK subsets across the three PBMC-based models of chronic infection. Each point corresponds to a population cluster that was identified by FlowSOM. (**A**) CD94^hi^ NKG2A^hi^ CD16^+^ NK cells. (**B**) CD16^+^ NK cells that highly expressed one or several ICP molecules (PD1, TIM3, TIGIT, Lag3). (**C**) ILC and NK cell subsets that expressed high levels of PDL1/PDL2: C18, C19 for LV (Fig. 3D); C4, C6, C19 for ART (Supplemental Fig. 2D); and C12, C19 for env^−^ (Supplemental Fig. 3D). Values are relative frequency for (A) and (B) to normalize values across experiments and absolute frequency for (C). Statistical analysis was performed by one-way ANOVA with Dunnett’s multiple comparison with the uninfected control using Prism 9. * *P* ≤ 0.05, ** *P* ≤ 0.01, *** *P* ≤ 0.001, ns *P* > 0.05.

The main phenotypic changes that were uncovered by the cluster analysis in one or more chronic infection models are summarized in Fig. 4. First, there was a reduced frequency of CD94^hi^ NKG2A^hi^ cytotoxic NK cells in two models of chronic infection (Fig. 4A). CD94/NKG2A is a heterodimeric inhibitory receptor that recognizes HLA-E on target cells. The cytotoxic functions of NK cells depend on a fine balance in the patterns of expression of inhibitory and activating receptors. Many confounding factors affect this balance in vivo, and HIV viremic patients with advanced clinical status have reportedly either increased or decreased levels of NKG2A expression on cytotoxic NK cells (45–47). The in vitro acute condition of this study may better mirror primary infection, in which no changes in NKG2A levels were reported (48). The decrease in NKG2A^hi^-expressing cells detected in the LV and ART chronic cocultures models suggests that NK cells might have increased cytotoxic capacity compared with env^−^ and acute infection models, and it will be interesting to identify the factors that underlie these differences.

Second, a reduced frequency of ICP^hi^ cytotoxic NK cells was also detected in the LV and ART coculture conditions (Fig. 4B). Similarly, to NKG2A, elevated levels of inhibitory receptors such as TIGIT, Lag3, and PD1 mediate NK cell immunosuppression and drive immune exhaustion in patients with HIV (49, 50). Here, the reduced frequency of NK cells expressing high levels of these inhibitory receptors in chronic models of HIV infection may point to an enhanced capacity to control virally transformed cells, although further work will be needed to uncover the mechanisms involved.

The unsupervised analysis also uncovered an enrichment in PDL1/PDL2^hi^ ILC and NK cell subsets, but only in the LV condition (Fig. 4C). The elevation of PD1 ligand-expressing cells might be expected to activate PD1 signaling in LV cocultures; however, this effect may be confounded by the concomitant decrease in PD1-expressing cells.

In conclusion, our study uncovered numerous phenotypic changes that are not attributed to direct HIV-1 infection but are triggered by coculture with infected cells in the absence of ongoing replication, suggesting that innate immune cells can sense persistently infected cells. Further work will be needed to identify the DAMPs and alarmins expressed by chronically infected cells and to test whether ICP inhibitors can restore innate immunity in infectious disease as is observed in cancer (51). In particular, ILCs are emerging as highly plastic cells that are wired to sense minute alterations in tissue homeostasis and swiftly regulate their functions and phenotypes in response to danger signals.

## Discussion

Despite successful ART, patients with HIV have chronic low-grade inflammation, which increases many comorbidities, such as diabetes, vascular disease, and an increased risk of cancer. The causes for this chronic inflammation are still being investigated. Cell-based models are a useful tool because they allow researchers to precisely control the experimental conditions of chronic infection (52), as well as to control the exposure of ILCs to persistently infected cells. They furthermore provide a versatile model to use pharmacologic or genetic approaches to test different agonistic or inhibitory effectors of ILC activation, thereby providing a unique opportunity to better understand, at the molecular level, how a dysfunctional innate immunity can sustain chronicity. In this study, we used primary cell-based models of persistent infection to uncover common patterns of dysfunction in the ILC compartment and possible mechanisms of action.

Although in vitro experiments offer a simplified version compared with patients, they are necessary to precisely control the induction of latency and the exposure of ILCs to persistently infected cells. Importantly, the use of in vitro coculture methodologies of ILCs and tumor cell lines has been successful in recapitulating the ex vivo observed ILC dysfunctions in cancer. For instance, ILC1 functional impairment (e.g., decrease in proinflammatory cytokine secretion) can be recapitulated in vitro by exposing healthy donor ILCs to melanoma cell lines (53). Also, defective ILC2 activities observed in patients with colorectal cancer were generated in vitro by coculturing ILC2s with colorectal cancer cell lines (54). Although these findings provided initial clues on a bidirectional cross-talk between tumor cells and ILCs, the phenotypic changes, the molecular pathways, and possible intermediate players involved in this interaction remain largely unexplored.

The design of the study has several limitations. The first is linked to the use of peripheral blood to study ILCs. These are quite rare in circulating blood and do not have the same phenotype as tissue-resident ILCs (41). In particular, the CD56^dim^ population predominates in blood (90% of NK cells). Moreover, although the uninfected ILCs that were analyzed at d9 stem predominantly from the fresh batch of autologous cells that was added at d6 and was never exposed to virus or to PHA, we cannot rule out that uninfected PHA-stimulated cells from d0 may still be present at d9 and therefore included in the analysis. Similarly, we cannot rule out that changes observed in the ART condition are not due to the drug itself rather than to suppressed viral replication. A second limitation is that our models of persistent HIV infection do not reproduce the transcriptionally latently infected reservoir, which is predominantly resting CD4^+^ T cells.

Negative regulatory receptors, also known as ICPs, are expressed on the surface of activated immune cells. Upon ligation of their cognate ligand, they transduce signaling cascades that fine tune their output by inhibiting effector functions. Although expression of ICPs on adaptive immune cells has been studied extensively and exploited for treatment, much less is known about inhibitory receptor expression in innate immune cells (55). Like in cancer, these ICPs may work to the advantage of latent viruses and confer immune evasion. In virally suppressed individuals, the expression of PD1, TIGIT, and LAG-3 is associated with CD4^+^ T cells harboring integrated proviruses (56). Moreover, ICPs were shown to contribute to reservoir persistence and to T cell exhaustion, and ICP blockade has been investigated as a latency reversal approach (57–60).

NKG2A is an inhibitory receptor expressed on the surface of NK cells as a heterodimer with CD94 (61). Its ligand, HLA-E, is expressed on normal cells and frequently overexpressed in many hematological and solid tumors, thus inhibiting NK cell functions. Compellingly, anti-NKG2A Abs enhance NK cell activity against various tumor cells and constitute a promising new immunotherapy approach (62, 63). In our cell-based systems, the enrichment of CD94^lo^ and NKG2A^lo^ NK cells in all models of chronic infection was somewhat unexpected because it suggests an activation of NK cell functions rather than inhibition. In fact, however, a similar downmodulation of NKG2A was recently reported in NK cells from patients with chronic hepatitis B (64). Similarly, although HLA-E is frequently upregulated on tumor cells, it is in fact downmodulated by many viruses, such as herpesviruses, influenza A and B, cowpox virus, and vaccinia virus, by evolutionarily acquired mechanisms to interfere with MHC class I presentation of viral peptides (65, 66). The suppression of endogenous MHC class I expression has the potential to render cells more vulnerable to NK cell attack. Therefore, the loss of CD94^+^ NKG2A^+^ cells that we observed may in fact point to an inability of NK cells to identify and kill infected cells.

Checkpoint molecules and ligands on helper ILCs are much less characterized than in NK cells. We observed an overall increase in the expression of key inhibitory receptors/ligands and ICP molecules in models of chronic infection. The engagement of PD1, primarily expressed on T cells, via its ligands PDL1 and PDL2 delivers an inhibitory signal to the PD1–bearing cell. Therefore, upregulation of PDL1 on ILC2 may lead to inhibitory control of adaptive T cell responses. Conversely, the upregulation of the inhibitory receptors 2B4 and TIGIT on ILC1s is likely to reflect an inhibition of ILC1 functions.

Intriguingly, these observations, at least in part, mirror a pattern of ILC dysfunctions observed ex vivo in patients with cancer. For instance, using flow cytometry panels similar to those presented above, NKG2A alterations have been reported in ILC1-like cytotoxic cells in acute myeloid leukemia, accounting for impaired killing capacity by these cells in patients (67). Increased expression of CD69 was detected in tumor-infiltrating ILCs in treatment-resistant patients (68). Although CD69 is generally considered as the earliest activation cell surface marker, recent evidence shows that CD69^+^ CD4^+^ T cell subpopulations represent potent immunosuppressive cells acting either by IL-10 or by TGF-β secretion (69, 70). Yet, nothing is known of the role of this molecule in ILCs. Furthermore, PDL1 is upregulated on ILCs in patients with hematologic malignancies, potentially fostering Th2 responses (71).

Our working premise is that in persistent infections, poor innate immune responses lead to ineffective immunity and an inability to clear the pathogen. Such in vitro cell-based models could help identify key components of the innate immune system that can be targeted to clear persistent infections. Although the disruptions incurred by the innate immunity during chronic infections have been examined for a few individual pathogens (72–74), we lack a comprehensive vision of common weaknesses that could be targeted to awaken innate immunity.

## Supplementary Material

Supplemental 1 (PDF)
