# Supplementary material for: Assessing the Impact of Persistent HIV Infection on Innate Lymphoid Cells Using In Vitro Models
Source: Immunohorizons. 2023 Mar 31;7(3):243–55. doi: 10.4049/immunohorizons.2300007 (PMC10563434; doi:10.4049/immunohorizons.2300007)
Supplement: Supplemental 1 (PDF) [file IH_2300007_Supplemental_1.pdf]

A

Laser	Fluorophore	Panel 1	Panel 2	Panel 3	Panel 4	Panel 5	Panel 6	
Blue 488nm	FITC	Dead	Dead	Dead	Dead	Dead	Dead	Pan-panel mix
		CD3	CD3	CD3	CD3	CD3	CD3	
		CD4	CD4	CD4	CD4	CD4	CD4	
		CD14	CD14	CD14	CD14	CD14	CD14	
		CD15	CD15	CD15	CD15	CD15	CD15	
		CD19	CD19	CD19	CD19	CD19	CD19	
		CD20	CD20	CD20	CD20	CD20	CD20	
		CD33	CD33	CD33	CD33	CD33	CD33	
		CD34	CD34	CD34	CD34	CD34	CD34	
		CD203c	CD203c	CD203c	CD203c	CD203c	CD203c	
		FceRI	FceRI	FceRI	FceRI	FceRI	FceRI	
	PerCP Cy5.5	CRTH2	CRTH2	CRTH2	CRTH2	CRTH2	CRTH2	
Red 633nm	APC	CD94	CD94	CD94	CD94	CD94	CD94	
	A700	CD16	CD16	CD16	CD16	CD16	CD16	
	APC Cy7	CD8	CD8	CD8	CD8	CD8	CD8	
Violet 405nm	BV421	CD127	CD127	CD127	CD127	CD127	CD127	
	BV605	cKit	cKit	cKit	cKit	cKit	cKit	
	BV785	CD56	CD56	CD56	CD56	CD56	CD56	
	BV510	CD25	2B4	NKG2D	OX40	CD86	ICOS	Panel-specific mix
	BV650	CD62L	Tim3	NKp46	CD28	CD80	CCR6	
	BV711	CD137	PD1	NKp30	PDL1	HLA DR	CXCR3	
Yellow 561nm	PE	KLRG1	CTLA4	NKp80	IFN-gR	IL-9R	OX40L	
	PE-Dazzle594	CD45RO	TIGIT	NKG2A	PDL2	CD40	HVEM	
	PE-Cy7	CD69	Lag3	NKp44	Galectin-9	CD70	CD137L	

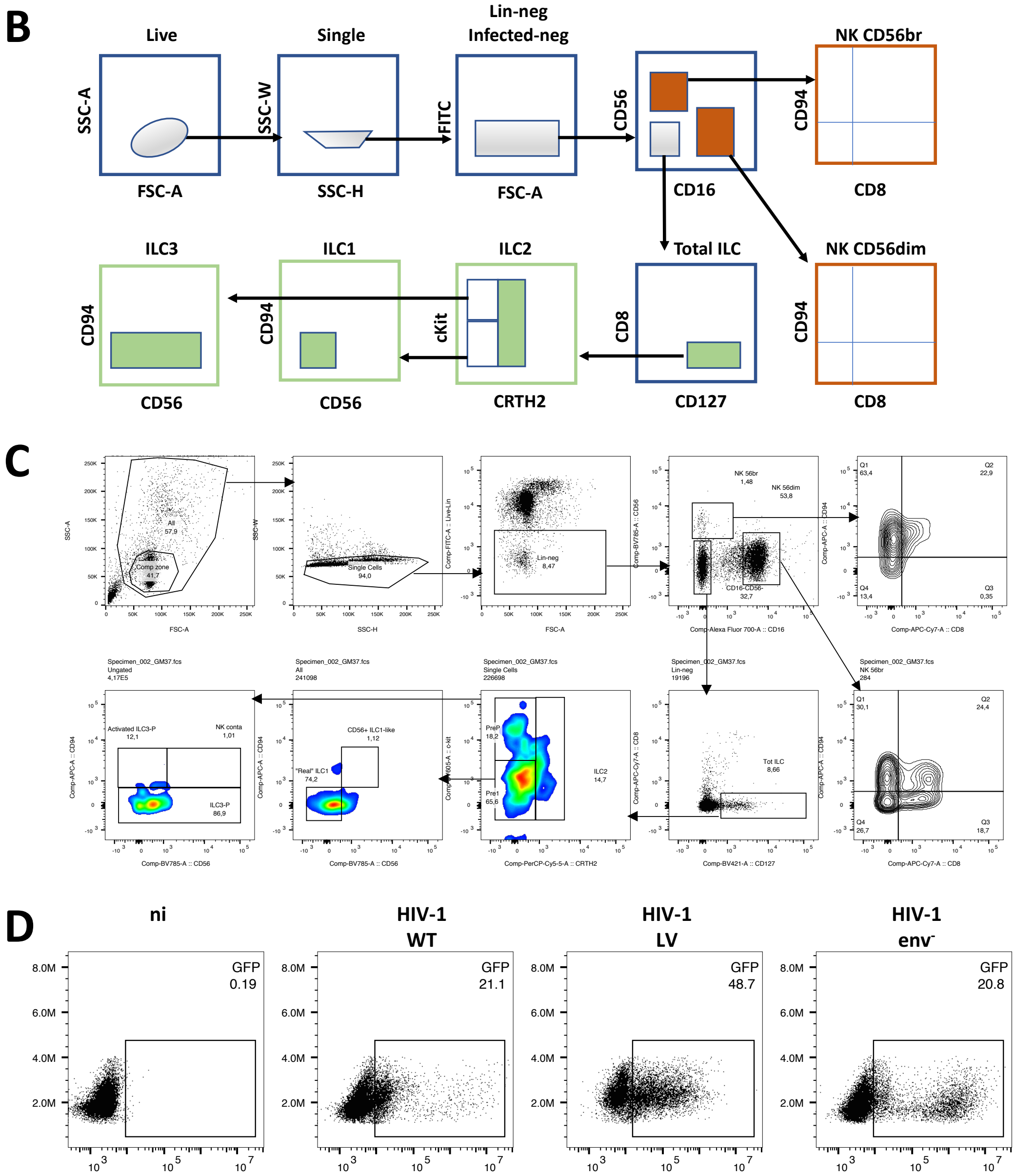

Suppl. Fig. 1: (A) Antibody panels that were used for labelling the co-cultures. **(B)** Human NK/ILC gating strategy. Immunoregulatory NK cells are CD16$^-$ CD56^{br}, cytotoxic NK cells are CD16^{br} CD56^{dim}. Total ILCs express high levels of the IL7 receptor (CD127), and are further subdivided on their expression of CRTH2 and cKit. **(C)** Representative flow cytometry plots showing the gating strategy. (**D)** Representative plots showing the efficiency of infection in human PBMCs by HIV-1 wild-type (WT), lentiviral vector (LV) and env-defective (env$^-$), at 3 days post-infection just prior to co-culture. The percentages of eGFP-positive cells are indicated in the top right corner.

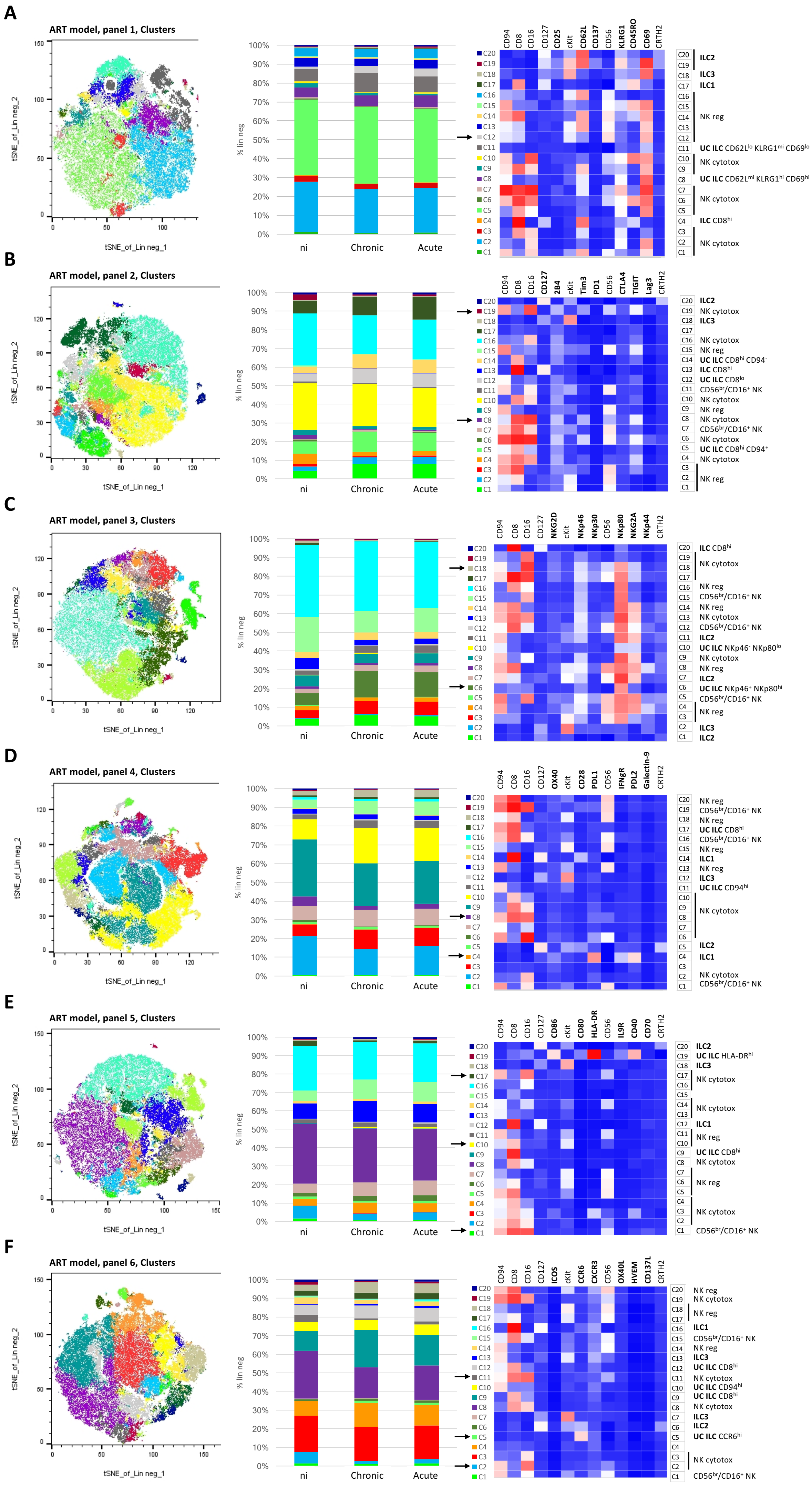

Suppl. Fig. 2: t-SNE dimensionality reduction analysis of PBMCs from the ART model co-culture. tSNE plots are coloured to indicate the 20 clusters and correspond to antibody panels (Suppl. Fig. 1A) 1 (**A**), 2 (**B**), 3 (**C**), 4 (**D**), 5 (**E**) and 6 (**F**). Clusters are quantified as % of Lin$^-$ and labelled by manual cell type annotation based on the gating strategy shown in Suppl. Fig. 1B. UC=Unconventional ILC (CD56$^-$CD16$^-$CD127$^-$); Unchar.=uncharacterized, negative for all tested markers. Clusters that are discussed in the results section are pinpointed by a black arrow.

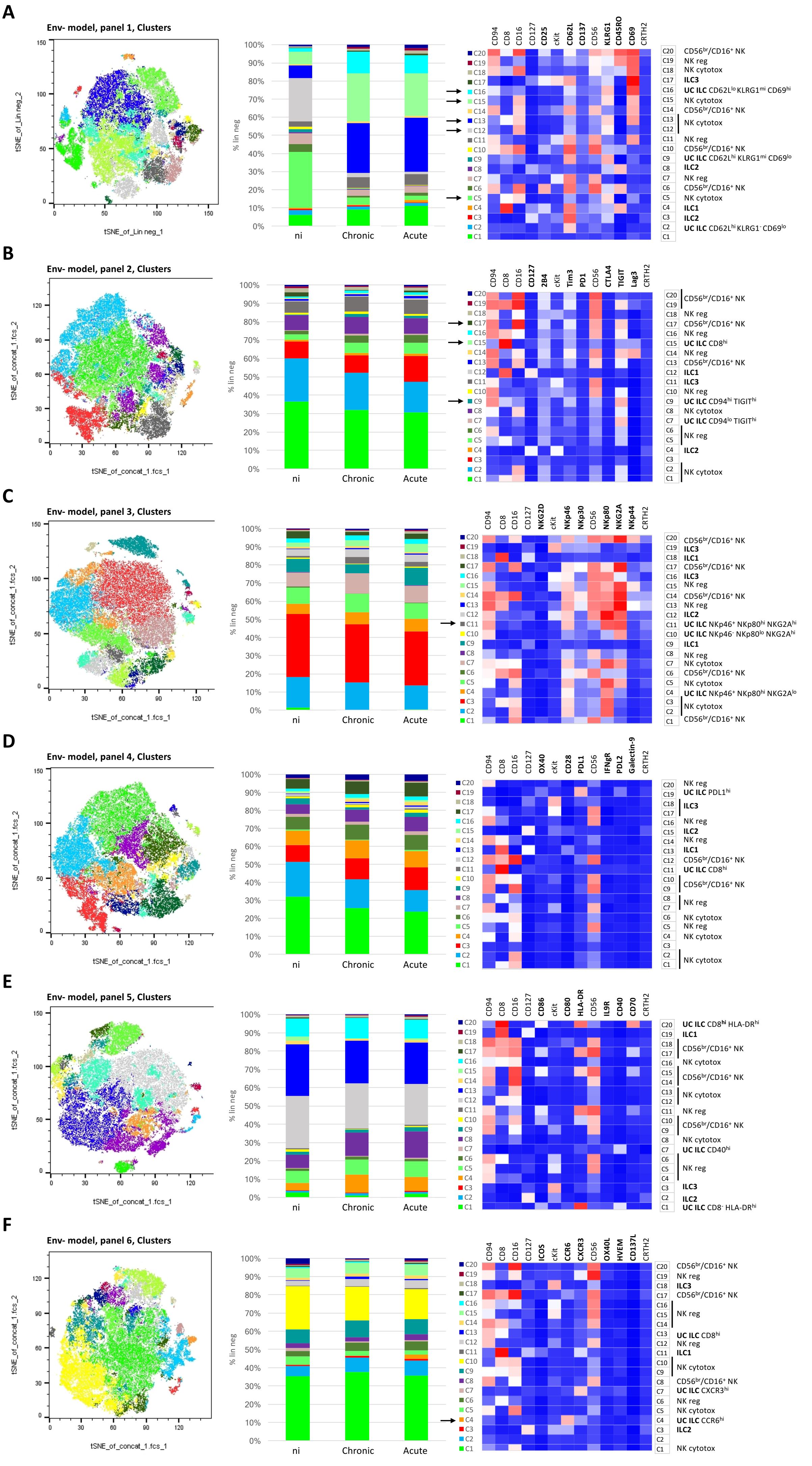

Suppl. Fig. 3: t-SNE dimensionality reduction analysis of PBMCs from the env⁻ model co-culture. tSNE plots are coloured to indicate the 20 clusters and correspond to antibody panels (Suppl. Fig. 1A) 1 (**A**), 2 (**B**), 3 (**C**), 4 (**D**), 5 (**E**) and 6 (**F**). Clusters are quantified as % of Lin⁻ and labelled by manual cell type annotation based on the gating strategy shown in Suppl. Fig. 1B. UC=Unconventional ILC (CD56⁻CD16⁻CD127⁻); Unchar.=uncharacterized, negative for all tested markers. Clusters that are discussed in the results section are pinpointed by a black arrow.

Suppl. Table 1: List of antibodies used for the study. The volumes correspond to the optimal stain index for each antibody. REC= recombinant antibodies obtained from mammalian cell cultures.

	Fluorochrome	Surface antigen	Supplier	Clone	Host	Reference	Lot	Volume (µl)	Use
1	FITC	CD3	Miltenyi Biotec	REA613	REC	130-113-138	5191024114	1	Basic panel & compensation
2	FITC	CD4	Miltenyi Biotec	REA623	REC	130-114-531	5191113548	1	Basic panel
3	FITC	CD14	Miltenyi Biotec	REA599	REC	130-110-518	5191115136	1	Basic panel
4	FITC	CD15	Biolegend	W6D3	Mouse	323004	B269981	2	Basic panel
5	FITC	CD19	Miltenyi Biotec	REA675	REC	130-113-645	5191028076	0,5	Basic panel
6	FITC	CD20	Miltenyi Biotec	REA780	REC	130-111-337	5191118118	1	Basic panel
7	FITC	CD33	Miltenyi Biotec	REA775	REC	130-111-018	5191118344	0,5	Basic panel
8	FITC Vio Bright B515	CD34	Miltenyi Biotec	REA1164	REC	130-120-517	5191111156	2	Basic panel
9	FITC	CD203c	Biolegend	NP4D6	Mouse	324614	B267681	2	Basic panel
10	FITC	FceRI	Miltenyi Biotec	REA758	REC	130-110-726	5191118099	2	Basic panel
11	PerCP-Cy5.5	CD294 (CRTH2)	Biolegend	BM16	Rat	350116	B266750	2	Basic panel
12	PerCP-Cy5.5	CD4	Miltenyi Biotec	REA623	REC	130-113-790	5191115133	0,5	Compensation
13	PE	KLRG1	eBiosciences	13F12F2	Mouse	12-9488-41	2056560	0,5	Panel 1
14	PE	CD152 (CTLA4)	Miltenyi Biotec	REA1003	REC	130-116-930	5191115142	2	Panel 2
15	PE	NKp80	Miltenyi Biotec	REA845	REC	130-112-779	5191118134	1	Panel 3
16	PE	IFN-gR CD119	Miltenyi Biotec	REA161	REC	130-100-013	5191118067	1	Panel 4
17	PE	IL-9R CD129	Biolegend	AH9R7	Mouse	310403	B291481	2	Panel 5
18	PE	CD252 (OX40L)	Biolegend	11C3.1	Mouse	326307	B274115	2	Panel 6
19	PE	CD3	Biolegend	OKT3	Mouse	317308	B292861	0,5	Compensation
20	PE/Dazzle594	CD45RO	Miltenyi Biotec	REA611	REC	130-114-088	5191118124	2	Panel 1
21	PE/Dazzle594	TIGIT	Miltenyi Biotec	REA1004	REC	130-116-936	5191114186	2	Panel 2
22	PE/Dazzle594	CD159a (NKG2A)	Miltenyi Biotec	REA110	REC	130-120-121	5191028096	0,5	Panel 3
23	PE-Vio 615	CD273 (PDL2)	Miltenyi Biotec	REA985	REC	130-116-691	5191115141	2	Panel 4
24	PE/Dazzle594	CD40	Biolegend	5C3	Mouse	334341	B280841	2	Panel 5
25	PE/Dazzle594	CD270 (HVEM)	Biolegend	122	Mouse	318817	B282921	2	Panel 6
26	PE-Vio 615	CD8	Miltenyi Biotec	REA734	REC	130-110-823	5191114786	2	Compensation
27	PE-Vio 770	CD69	Miltenyi Biotec	REA824	REC	130-112-615	5191203388	2	Panel 1
28	PE-Cy7	CD223 (Lag3)	Biolegend	11C3C65	Mouse	369309	B289009	0,5	Panel 2
29	PE-Vio 770	CD336 (NKp44)	Miltenyi Biotec	REA1163	REC	130-120-366	5191118156	0,5	Panel 3
30	PE-Cy7	Galectin-9	Biolegend	9M1-3	Mouse	348915	B237280	0,5	Panel 4
31	PE-Cy7	CD70	Biolegend	113-16	Mouse	355111	B278367	0,5	Panel 5
32	PE-Vio 770	CD137L	Miltenyi Biotec	REA254	REC	130-119-153	5191118151	2	Panel 6
33	PE-Vio 770	CD3	Miltenyi Biotec	REA613	REC	130-113-702	5191118119	2	Compensation
34	APC	CD94	Miltenyi Biotec	REA113	REC	130-098-976	5191118059	1	Basic panel
35	AF647	CD4	Miltenyi Biotec	REA623	REC	130-113-784	5191118306	0,5	Compensation
36	A700	CD16	Biolegend	3G8	Mouse	302026	B266048	0,5	Basic panel
37	A700	CD3	Biolegend	SK7	Mouse	344821	B309642	2	Compensation
38	APC-Cy7 (Vio 770)	CD8	Miltenyi Biotec	REA734	REC	130-110-681	5191111661	1	Basic panel & compensation
39	BV421	CD127	Biolegend	A019D5	Mouse	351310	B279332	0,5	Basic panel
40	BV421	CD3	Biolegend	UCHT1	Mouse	300433	B278035	2	Compensation
41	BV510	CD25	Biolegend	BC96	Mouse	302639	B285257	2	Panel 1
42	BV510	CD244 (2B4)	Biolegend	C1.7	Mouse	329533	B291936	1	Panel 2
43	BV510	CD314 (NKG2D)	Biolegend	1D11	Mouse	320815	B281317	0,5	Panel 3
44	BV510	CD134 (OX40)	Biolegend	Ber-ACT35	Mouse	350025	B292967	1	Panel 4
45	BV510	CD86	Biolegend	IT2.2	Mouse	305431	B275091	0,5	Panel 5
46	BV510	CD278 (ICOS)	Biolegend	C398.4A	enian har	313525	B269041	2	Panel 6
47	BV510	CD4	Biolegend	OKT4	Mouse	317443	B277492	1	Compensation
48	BV605	CD117 (cKit)	Biolegend	104D2	Mouse	313218	B284316	2	Basic panel
49	BV605	CD8	Biolegend	SK1	Mouse	344741	B277535	2	Compensation
50	BV650	CD62L	Biolegend	DREG-56	Mouse	304831	B277549	1	Panel 1
51	BV650	CD366 (Tim3)	Biolegend	F38-2E2	Mouse	345027	B288730	2	Panel 2
52	BV650	CD335 (NKp46)	Biolegend	9E2	Mouse	331927	B270439	1	Panel 3
53	BV650	CD28	Biolegend	CD28.2	Mouse	302945	B264446	2	Panel 4
54	BV650	CD80	Biolegend	2D10	Mouse	305227	B263645	2	Panel 5
55	BV650	CD196 (CCR6)	Biolegend	G034E3	Mouse	353425	B251357	1	Panel 6
56	BV650	CD3	Biolegend	UCHT1	Mouse	300467	B290426	2	Compensation
57	BV711	CD137	Biolegend	4B4-1	Mouse	309831	B253102	2	Panel 1
58	BV711	CD279 (PD1)	Biolegend	EH12.2H7	Mouse	329927	B286178	2	Panel 2
59	BV711	CD337 (NKp30)	Biolegend	P30-15	Mouse	325218	B292257	2	Panel 3
60	BV711	CD274 (PDL1)	Biolegend	29E.2A3	Mouse	329721	B249259	2	Panel 4
61	BV711	HLA DR	Biolegend	L243	Mouse	307643	B275035	2	Panel 5
62	BV711	CD183 (CXCR3)	Biolegend	G025H7	Mouse	353731	B230632	2	Panel 6
63	BV711	CD3	Biolegend	SK7	Mouse	344837	B261313	2	Compensation
64	BV785	CD56	BD Biosciences	MY31	Mouse	742662	9337099	2	Basic panel
65	BV785	CD3	Biolegend	SK7	Mouse	344841	B222704	2	Compensation
